# Intimate Partner Violence Against Men in Germany—A Study on Prevalence, Victim–Offender Overlap, and the Role of Parental Violence

**DOI:** 10.1177/08862605251321003

**Published:** 2025-02-26

**Authors:** Jonas Schemmel, Dario Maciey, Laura-Romina Goede

**Affiliations:** 1 FernUniversität in Hagen, Germany; 2 Psychologische Hochschule Berlin, Germany; 3 Criminological Research Institute of Lower Saxony, Hannover, Germany

**Keywords:** intimate partner violence against men, IPV, parental violence, childhood violence, victim–offender overlap, prevalences

## Abstract

We present data on intimate partner violence (IPV) victimization, perpetration, and victim–offender overlap in Germany, focusing on the impact of parental violence. We collected a sample using a register-based procedure where 183 randomly selected municipalities provided the addresses of 12,000 randomly selected men aged 18 to 69. Out of these men, a total N of 1,209 answered questions on their experiences with IPV and parental violence as children. In our sample, lifetime prevalences of IPV victimization ranged from 5.4% (sexual violence) to 39.8% (psychological violence), and 12-month prevalences ranged from 2.8% (digital violence) to 25.1% (coercive control). The sample’s corresponding lifetime prevalences of IPV perpetration ranged between 2.3% (digital violence) and 33.4% (psychological violence). Overall, there was a victim–offender overlap of 39.5%, which was particularly pronounced for non-physical IPV (psychological: 23.6%; coercive control: 20.3%). Offending only was most frequently reported for coercive control (18.4%). Across the different IPV types, victimization was consistently associated with verbal parental violence in childhood. Being victimized by verbal parental violence and witnessing violence between parents were predictive of later being involved in psychological violence as a victim-only or as both a victim and offender. Being a victim of physical parental violence in childhood more than doubled the odds of being a victim–offender of physical IPV, and increased the odds of becoming an offender-only of sexual IPV. These findings suggest that distinguishing clearly between victim and offender is often challenging in IPV research and reaffirm the well-established link between parental violence and IPV in the context of IPV against men.

Intimate partner violence (IPV) against men has received growing scientific interest in recent years, specifically regarding the influence of sex and gender identity on victims’ perceptions of violence and their patterns of seeking help (for a comprehensive overview, see Scott-Storey et al., 2022). Research has stressed societal factors that contribute to an underrepresentation of men as victims as they often struggle to self-identify as victims and to assess their risk of harm due to internalized male stereotypes (Hine, Bates, et al., 2022; Lysova et al., 2022). Also, society usually perceives men as perpetrators of IPV rather than as victims which results in their reluctance to openly address their IPV experiences or even report them to the police as they fear discreditation and a loss of status (Hine, Wallace, et al., 2022; Lysova et al., 2020; Taylor et al., 2022). This may have contributed to a general lack of large, comprehensive data sets on male IPV experiences. This study reports data from a survey using representative methods on various types of IPV involvement of men in Germany. Specifically, we focus on the victim–offender overlap (Park & Kim, 2019), and parental violence as an important risk factor for men’s later IPV involvement (Varlioglu & Hayes, 2022).

## IPV against Men—Prevalence

Nowinski and Bowen (2012) compiled 34 studies focusing on IPV against men, of which only some were based on sufficiently large or representative samples from the general population. These studies reported overall sample prevalences (of all forms of violence) for heterosexual men between 7.3% and 32.4% (lifetime prevalence) and between 0.6% and 29.3% (12-month prevalence). Sample lifetime prevalences for physical violence ranged from 7.4% to 54.0% (12-month prevalence: 0.9%–28.6%), for psychological violence from 18.3% to 93% (12-month prevalence: 30.2%–87.0%), and for sexual violence from 0.2% to 0.3% (12-month prevalence: 15.4%, only one prevalence number reported). These rates were generally lower in large-scale studies with bigger samples and when violence was assessed using the Conflict Tactics Scale (CTS). However, not all studies made a clear distinction among various forms of violence. Particularly noteworthy is the infrequent separate reporting of psychological and, even more, of sexual violence. In a somewhat more recent but less extensive review, Kolbe and Büttner (2020) reported IPV prevalence figures from 17 studies, presumably referring to the participants’ lifetimes. Overall, the prevalences for physical IPV against men with no handicaps ranged from 3.4% to 20.3%, for psychological IPV from 7.3% to 37.0%, and for sexual IPV from 0.2% to 7.0%.

Recently, Jud et al. (2023) presented the first nationwide representative study on IPV against men in Germany. Their study focused on IPV against both men and women and was published after the current data collection was finished. Overall, data from 1,347 men with a minimum age of 14 years was collected using a representative sampling method. Based on the German translation of an international questionnaire on psychological (five items), physical (three items), sexual (four items), and economic (three items) IPV (Jewkes et al., 2017), they presented lifetime prevalences and ordinal scale reports (rarely, occasionally, regularly). Overall, 50.8% of the men reported at least one violent incident, with 48.0% reporting at least one act of psychological violence, 10.8% at least one act of physical violence, 7.5% at least one act of economic violence, and 5.5% at least one act of sexual violence. Various forms of violence consistently co-occurred, with psychological violence exhibiting the highest frequency of overlap with other types of violence. IPV victimization was associated with older age, poverty, unemployment, and IPV perpetration. Women reported consistently higher frequencies across all violence types; the largest difference occurred regarding sexual violence (18.6% vs. 5.5%).

## Victim–Offender Overlap and IPV

The victim–offender overlap is a well-known criminological phenomenon. It states that individuals who have been victims of crimes often commit crimes themselves, and offenders frequently experience victimization (Berg & Schreck, 2022; Lauritsen & Laub, 2007). This finding holds across various countries and for a multitude of different offenses (see Jennings et al., 2012). According to Tillyer and Wright (2013), exploring the victim–offender overlap is particularly relevant for understanding IPV. This is because intimate partners likely come together due to similarities in interests, personality, and social circumstances—factors discussed as potential reasons for the overlap (see the preceding paragraph). Furthermore, it is assumed that partnership stress, partly influenced by external factors, leads to conflicts that are not easily resolved, thereby fostering additional conflict situations. In a review of IPV against men, Kolbe and Büttner (2020) found that many affected men had engaged in domestic violence, and many violent women had themselves experienced IPV (see also Swan et al., 2012). Richards et al. (2016) even stated that mutual IPV is the most common type of IPV.

However, only a few studies specifically investigated the victim–offender overlap in IPV. Tillyer and Wright (2013) analyzed data from The National Longitudinal Study of Adolescent Health, a long-term survey of adolescents in grades 7 through 12 in the United States at various time points. Overall, approximately one-fifth (20.6%) of young men were involved in violence within partnerships. Among these, 13.8% were exclusively victims of partner violence, 1.1% exclusively acted as perpetrators of partner violence, and 5.7% were both victims and perpetrators of partner violence. Varlioglu and Hayes (2022) investigated physical dating violence in youth and young adulthood (college sample, 18–25 years, 12-month prevalences) and adverse childhood experiences (i.e., witnessing violence, victimization) using data from the International Dating Violence Study. This revealed that approximately 6% of the n=1060 surveyed men reported only experiencing partner violence, while 3% reported only perpetrating it. Approximately, a quarter (23.9%) of men were both perpetrators and victims.

Clemens et al. (2023) published a secondary analysis of the data presented by Jud et al. (2023) on the overlap of IPV victimization and perpetration in Germany. They identified a comparatively large victim–offender overlap in men for psychological violence (41.2% overlap vs. 10% victimization only). For the remaining forms of violence, the overlap rates were lower than the victimization frequencies, and the prevalence rates were substantially lower overall, although the overlap rates remained substantial (Physical: Overlap = 2.5%, Victimization = 5.8%; Economic: Overlap = 2.3%, Victimization = 8.8%; Sexual: Overlap = 3.1%, Victimization = 9.5%). Overall, the overlap was slightly higher for men than for women, particularly for sexual violence, where being both a victim and a perpetrator was reported almost as frequently as victimization alone (2.8% vs. 3.3%), and the prevalence of perpetration alone was markedly higher (9.1%). Furthermore, the analysis revealed that, overall, men reported more IPV perpetration while women reported more IPV victimization.

## Violent Childhood Experiences as Risk Factor for IPV Against Men

Experiencing violence in the family during childhood is an important risk factor for IPV experiences later in life (Costa et al., 2015; Li et al., 2019; Milletich et al., 2010). Among the many explanations discussed for the association of IPV with violent childhood experiences (for overviews see Ali & Naylor, 2013; Chester & DeWall, 2018), social learning theories (SLT) are arguably among the most popular. They posit that children from violent families internalize a violence-prone model that shapes their own later behavior in relationships (Forke et al., 2018; Varlioglu & Hayes, 2022). In other words, children witnessing violence between parents or against themselves may adopt their conflict resolution strategies (imitation) perhaps through operant conditioning or observational learning (Holt et al., 2008). While social learning theories tend to focus on IPV perpetration, some works suggest that they can also explain why parental violence increases the likelihood of later IPV victimization (Hamby et al., 2012). For example, SLT points to socialization processes such as differential association (e.g., with other victims of parental violence showing attitudes that excuse violence or suggest self-blame for violent experiences). In general, children from violent households may socialize in violence-supporting or violence-excusing environments in and outside the family, which not only increases the risk of exposure to a violent partner later in life but also increases the tendency to normalize violent behavior in relationships (Powers et al., 2020). Indeed, studies have shown that both being victimized by parents and witnessing violence between parents predict attitudes justifying IPV, especially among men (Kinsfogel & Grych, 2004; Manchikanti Gomez, 2010). Such attitudes may cause one to trivialize violence by one’s own partner or lead one to perpetrate IPV.

However, it must be stressed that social learning—or more general: lifetime experience—is not the only mechanism that may explain associations between experiencing parental violence and later IPV (Widom, 1989). Since children and their parents share a large amount of genetic variance, and aggression in general has been shown to be influenced by genetic dispositions and mechanisms (Anholt & Mackay, 2012), it is plausible to assume that genetics play a role in IPV as well (Hines & Saudino, 2004). Indeed, research has found that genetic factors are important contributors to IPV perpetration (Barnes et al., 2013; DeWall & Way, 2014). Barnes et al. (2013) also showed that non-shared environmental factors explained all IPV variance not explained by genetics. Thus, combining both genetic and environmental factors may be important to understand the link between violence childhood experiences and IPV.

Varlioglu and Hayes (2022) correctly pointed out that most studies on IPV and child maltreatment do not consider victim–offender overlap, only distinguishing between victims and perpetrators. They found a significant effect of adverse socialization (pro-violent messages, witnessing violence during childhood, and experiencing violence that was not child maltreatment), neglect, and physical abuse in childhood on being a victim only of IPV within the male subsample (n=1060). No predictive effect was found for being an IPV offender-only or being both victim and offender of IPV. As these results differed from those found when only females were included in the analysis, one of the authors’ main conclusions was that there was a need for more gender-specific studies on the three types of IPV involvement and violent childhood experiences.

In their study on the victim–offender overlap in a representative German sample, Clemens et al. (2023) found that child maltreatment, measured with the Adverse Childhood Experiences Questionnaire (Felitti et al., 1998), increased IPV involvement of all sorts (victim, offender, victim–offender) for all types of IPV. However, they did not report separate analyses for different genders.

Nowinski and Bowen (2012) and Kolbe and Büttner (2020), on the other hand, identified adverse (i.e., violent) childhood experiences as important risk factors for IPV specifically against men, which underscores the importance of early violent experiences to enhance our understanding of men’s involvement in IPV.

## The Current Study

Using representative sampling methods, the current study presents data from a large study in Germany on the IPV experiences of men. It had three primary objectives: First, to contribute to the existing literature by offering insights into both lifetime and 12-month prevalence rates of five distinct types of violence (physical, psychological, sexual, economic, and coercive control) in Germany. Second, to investigate not only victimization but also offending, our aim was to investigate the victim–offender overlap in IPV for men. Third, to scrutinize the associations between violent childhood experiences and being a victim, an offender, or both. Our emphasis was on parental victimization and witnessing violence between parents, as these are well-researched risk factors that still require a deeper understanding.

## Methods

The data used for the reported analyses were collected as part of a larger study at the Criminological Research Institute of Lower Saxony, Germany, that included both quantitative and qualitative components surrounding the experiences of German men aged 18 to 69 years with IPV. Before data collection started, a comprehensive data protection procedure was created and approved by an independent data protection officer. The study was also approved by the ethics committee of the German Society of Psychology on July 4, 2022. Here, we only report the data necessary to address the specific objectives of the current study; a comprehensive overview of the research project is available as an open-access e-book, albeit in German (Schemmel et al., 2024). The data are not publicly available. Upon reasonable request, however, the authors are willing to share them using a data use agreement.

### Participants

#### Sampling Procedure

To aim for the representativeness of the data, a register-based sample was drawn. This involved a two-stage process. First, 183 municipalities from across Germany were selected, and then the number of participants to be drawn from each municipality was calculated.^1^ Subsequently, the selected municipalities were contacted, randomly chose the algorithm-calculated number of individuals (men aged 18 to 69) and transmitted their addresses so that they could be contacted via mail. The targeted total sample size to be drawn was 12,000 men aged 18 to 69. Overall, the addresses of 11,733 men were transmitted by the municipalities. These men were contacted by mail and invited to participate in an online survey from October 20, 2022, to February 20, 2023. The invitation did not mention IPV but more generally referred to conflicts and their handling in relationships. A reminder was sent two weeks later. An incentive was offered, with the announcement that 1 Euro would be donated to a charitable organization for every completed survey. Some individuals (18 total) responded by phone or email, expressing objection to the use of their address data. In addition, 410 letters were returned to the sender as they could not be delivered (e.g., due to relocation or the death of the addressee). Therefore, the gross sample size was *N* = 11,305.^2^

The survey was started by 1,489 individuals. The data cleaning focused on excluding cases with illogical values or combinations (e.g., one’s own age, duration of the relationship) and inconclusive, open-ended responses categorized as “other.” We also removed those participants who had not answered at least the sociodemographic, and relationship status questions. In the end, 1,209 questionnaires were eligible for analysis. Thus, the response rate was 10.7%.

#### Sample Characteristics

The average age of the surveyed men was 45.8 years (*SD* = 14.1), ranging from 18 to 70 years. The majority (92.9%) were born in Germany, and 15.4% had a migration background. Regarding the participants’ educational attainment, 40.3% held a degree from a university or a university of applied science, 25.4% completed high school, 23.0% completed middle school, and 7.9% left school after the eighth or ninth grade. The remaining 3.4% were still in school (0.3%), had left school without a degree (0.7%), or indicated a degree not listed among the choices provided (2.4%). In terms of income distribution, 8.1% earned less than 1,000 euros per month, 18.9% earned between 1,000 and 1,999 euros, 28.9% earned between 2,000 and 2,999 euros, 20.3% earned between 3,000 and 3,999 euros, 11.1% earned between 4,000 and 4,999 euros, and 12.7% earned more than 5,000 €. Approximately 70% were fully employed, 8.7% were partly employed, 13.0% were retired and 6.5% were unemployed. The remaining 2.6% were either in parental leave (0.3%), in training (2.1%), or doing military/social services (0.2%). Concerning relationship status, 80.1% were in committed relationships, with 59.1% married, 31.5% unmarried, 6.4% divorced, 0.6% widowed, 0.9% partnered. A significant majority (91.7%) had had at least one previous serious relationship, enabling responses to IPV-related questions. Thus, the demographic characteristics of our sample regarding age, migration, employment status, and relationship status were largely similar to those of the sample drawn by the German General Social Survey (restricted to 18–69-year olds, *n* = 2,075), a large representative survey in Germany conducted on a regular basis (GESIS—Leibniz-Institut für Sozialwissenschaften, 2023).^3^ However, men with a university degree as the highest level of education were slightly overrepresented in our sample (40.3% vs. 35.5%), and men having left school after the nineth grade were slightly underrepresented (7.9% vs. 13.9%). Men in our sample also reported a 4% to 5% larger share in all three top income categories, but a lower share in the low-medium category (1,000–1,999 €; 18.9% vs. 27.7%). This—along with the low response rate of 10.8%—may restrict the sample’s representativeness.

### Variables

IPV victimization and offending were analyzed as the most important (dependent) variables. The respondents with relationship experiences were asked about their experiences with various acts of physical violence (11 items), psychological violence (10 items), sexual violence (six items), coercive-control behavior (seven items), and digital violence (four items). The specific questionnaire was developed based on item catalogs from two studies conducted in Germany (Jungnitz et al., 2007) and Austria (Kapella et al., 2011). All forms contained an open-answer item “other” to check whether important acts had not been listed. As participants rarely answered this item and if they did, largely reported actions equivalent to those already listed, “other” was not included in the analyses. We provide all items in the Supplemental Appendix of this paper. For each act of violence, participants were initially asked to indicate the lifetime prevalence^4^ of victimization (*yes*/*no*) and perpetration (*yes*/*no*). If a respondent reported being affected by a specific act during their lifetime at least once, the 12-month prevalence was then assessed using a more fine-grained rating scale (never, 1 or 2 times, 3 to 12 times, multiple times per month, once per week, multiple times per week, daily). The 12-month prevalence of perpetration was not assessed because the primary focus of the overall project was on victimization and because we did not want to overload the questionnaire.

The key independent variable was parental violence. Specifically, we assessed whether participants had witnessed violence between their parents in their childhood and their own victimization by parental violence. *Victimization by parents* was assessed based on the conflict tactics scales by (Straus, 1979, 1990). It consisted of three items on non-physical violence (“My mother and/or my father screamed at me; verbally abused me; threatened to kick me out”) and six items on physical violence (“My mother and/or my father handled me roughly or pushed me; physically assaulted me; slapped me; threw an object at me; hit me with an object; hit me with their fist or kicked me”). All items were rated on a seven-point frequency scale (never to daily). The internal consistency of the scales was acceptable for non-physical violence (alpha = .77) and good for physical violence (alpha = .84). *Witnessing violence between parents* was assessed based on a scale by Wetzels (1997) and Wetzels et al. (2001), which had itself also been based on the conflict tactic scales by Straus (1979, 1990). It contained six items (“One parent threatened the other with divorce”; “My parents insulted each other”; “I witnessed my parents having loud arguments”; “My parents shouted at each other in my presence”; “I witnessed one parent forcefully pushing or shaking the other”; “I saw one parent beating the other”). Again, all items were rated on a seven-point frequency scale (never to daily). The internal consistency of the scale was good (alpha = .85).

Additional demographic items used for this article were age, education, income in euros per month, and migration background. Attained education was a multiple-choice question, with still in school/left school without completing the degree as the lowest possible educational attainment and a university degree as the highest. Income per month (after taxes) was assessed using a multiple-choice question with six income levels (“less than 1,000 €”; “1,000 to just under 2,000 €”; “2,000 to just under 3,000 €”; “3,000 to just under—4,000 €”; “4,000 to just under—5,000 €”; “more than 5,000 €”). Migration background was assessed as a binary variable, with migration background defined as either not having been born in Germany or having at least one parent who was not.

### Data Analysis

The data were analyzed with the statistical software SPSS 29 (IBM Corp., 2023). We report lifetime and 12-month prevalence rates of the sample for each type of violence. The 12-month prevalence rates are also reported using the original frequency ratings. The dichotomous frequencies of the different IPV involvement types (victim, offender and victim–offender) were calculated based on the lifetime prevalences. We conducted multinomial logistic regression analyses with involvement in IPV (victim-only, offender-only, victim and offender) as the dummy-coded dependent variable and parental violence (witnessing violence between parents, verbal parental violence, physical parental violence) as predictors for overall IPV and each type of IPV.^5^ Relevant demographic variables were entered as control variables (age, income, attained education, migration background). No IPV involvement was the reference category. Only digital violence was not analyzed separately due to small cell sizes that made inferential testing seem too unreliable (e.g., *n*_offender_ = 5). The type-I error threshold for all inferential analyses was set to 5%.

## Results

### Prevalences of IPV Against Men and Perpetrated by Men

**Figure 1.**
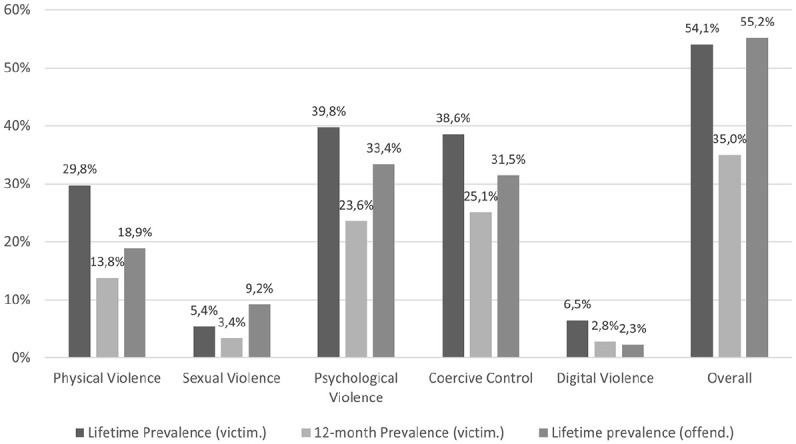
Lifetime (victimization and offending) and 12-month (victimization) prevalences (per IPV type and overall).

Figure 1 presents the lifetime and 12-month prevalences of victimization and the lifetime prevalence of perpetration by IPV type found in the sample. The highest lifetime victimization rate was observed for psychological violence (39.8%), followed by coercive control (38.6%), physical violence (29.8%), digital violence (6.5%), and sexual violence (5.4%). Over half of the respondents (54.1%) had experienced at least one type of IPV. Regarding the 12-month prevalence, the highest victimization rate was found for coercive control, with approximately a quarter of respondents (25.1%) having experienced it in the last 12 months. This was followed by psychological violence (23.6%), physical violence (13.8%), sexual violence (3.4%), and digital violence (2.8%). Overall, 35.0% of the surveyed men had been affected by at least one act of IPV in the last 12 months. Regarding perpetration, one-third of respondents (33.4%) engaged in psychological violence, and a similar proportion (31.5%) exercised coercive control toward their partner. The third-highest perpetration rate was found for physical violence (18.9%), followed by sexual violence (9.2%) and digital violence (2.3%). Over half of the respondents (55.2%) were perpetrators of IPV at least once in their lifetime.

Tables 1 and 2 present more detailed results regarding the most frequently reported violent acts of each IPV type. Note that the most severe acts of all IPV types are not included in the table as they occurred only very rarely across all IPV types. Also, an inspection of the frequency ratings regarding the last 12 months reveals that most men experienced violent acts only sporadically, and very few seemed to be victimized on a regular basis. More information on the prevalences of each IPV item is provided in the Supplemental Appendix of this article.

**Table 1.** Lifetime (Victimization and Offending) and 12-Month Prevalences (Victimization) With Frequency Rates Regarding the Last Year for the Two Most Frequently Reported Violent Acts Per IPV Type.

IPV Type	Violent Act	Lifetime (victim.) (%)	Lifetime (offend.) (%)	12 months (victim.) (%)	1–2 times (%)	3–12 times (%)	Muliple times a month (%)	At least once a week* (%)
Physical violence	Intentionally pushing	17.6	9.9	8.7	6.3	2.0	0.1	0.3
	Throwing an object	13.3	4.7	4.8	3.4	1.1	0.1	0.1
Psychological violence	Aggressively shouting, abusing, or insulting the other person	33.9	27.1	20.1	10.7	5.7	2.6	0.1
	Humiliating, devaluing, or belittling the other person	17.0	10.4	9.7	4.1	3.3	1.3	0.1
Sexual violence	Touching the other person with sexual intent^a^	4.6	8.4	2.5	1.6	0.7	0.1	0.1
	Forcing the other person to have sex/intercourse^b^	1.3	0.6	0.9	0.6	0.3	0.0	0.1
Coercive control	Blaming the other person for everything and constantly making the other person feel guilty	23.4	10.6	14.7	3.8	5.6	3.3	0.2
	Massive intimidation if the other person disagrees (e.g., through gestures, looks, or shouting)	17.2	10.3	9.2	3.9	3.5	1.0	0.8
Digital violence	Intimidating, blackmailing, or threatening through emails or text messages	4.2	0.8	1.8	0.6	0.5	0.6	0.2
	Spreading personal information or nasty rumors on social networks/the internet	3.2	0.1	1.2	0.5	0.5	0.1	0.2

*Note.* *To make the table more concise, the categories “*once a week*,” “*multiple times per week*,” and “*daily*” were combined as “*at least once a week*.” The frequencies of each of these categories were extremely low.

a The full wording of the item was: “Touching the other person with sexual intent (e.g., kissing, fondling, grabbing) even though the person has said or indicated that they do not want it.”

b The full wording of the item was: “Forcing the other person to have sex/intercourse even though the person has said or indicated that they do not want to.”

**Table 2.** Relative Frequencies of Reporting Victimization Only, Offending Only, Both Victimization and Offending, and No Involvement for Each Violence Type.

	Overall* (*n* = 1.076) (%)	Physical Violence* (*n* = 1.074) (%)	Psychological Violence* (*n* = 1.082) (%)	Sexual Violence* (*n* = 1.067) (%)	Coercive Control* (*n* = 1.095) (%)	Digital Violence* (*n* = 1.085) (%)
Victim	12.5	16.0	16.2	3.1	11.2	1.8
Offender	14.3	5.4	9.8	6.8	18.4	5.9
Victim–offender	39.5	13.5	23.6	2.3	20.3	0.6
No involvement	33.6	65.1	50.4	87.7	50.1	91.8

*Note.* The columns contain participants who reported at least one experience with one act of violence overall or of the respective type; percentages refer to all participants within one column.

* With *p* < .001, a chi-square test of independence indicated statistically significant differences between the proportions (calculations run without “no involvement”).

### Victim–offender Overlap in IPV Against Men

The similarly high sample prevalences of IPV victimization and perpetration presented above already indicate a potential overlap between perpetrators and victims. Table 1 presents relative percentages of being victimized by IPV, being an offender of IPV, the overlap of both, and how many participants reported no involvement in IPV. Across all IPV types, a significant proportion of the respondents reported an overlap of victimization and perpetration (39.5%). It far exceeded those of being only a victim (12.5%) or offender (14.3%).

A separate inspection of the IPV types reveals that this was probably because the share of being *victim and offender* was larger than the victim- and offender-only rates for the most frequently reported IPV types (psychological violence [23.6%] and coercive control [20.3%]) and lower than the victim- and offender-only rates for the least frequently reported IPV type (digital violence [0.6%]). It was substantial for physical violence (13.5%) and sexual violence (2.3%), but these values were still slightly lower than the victim-only rates (16% and 3.1%, respectively).

*Victim-only rates* were larger than offender-only and victim–offender rates regarding physical (16.0% vs. 5.4% and 13.5%). and larger than offender-only rates regarding psychological violence (16.2% vs. 9.8%). For the least frequent IPV types (sexual and digital violence), it was the other way around, that is, offender-only rates were higher than victim-only and victim–offender rates (sexual violence: 6.8% vs. 3.1% and 2.3%; digital violence: 5.9% vs. 1.8% and 0.6%). Therefore, although there were substantial victim–offender overlaps for all IPV types, there was a slight focus on victimization regarding physical and psychological violence. The victim-only rate of coercive control was considerably lower than the offender-only and the victim–offender rate (11.2% vs. 18.4% and 20.3). Therefore, o*ffender-only rates* were larger than victim-only rates regarding sexual violence (6.8% vs. 3.1%), coercive control (18.4% vs. 11.2%) and digital violence (5.9% vs. 1.8%). Among these IPV types, coercive control was the only one with a larger victim–offender overlap than victim- and offender-only rates (20.3% vs. 11.2% and 18.4%). Thus, the prevalences of sexual and digital violence showed a clear tendency toward offending, while the identified offending focus of coercive control was less pronounced.

### Victimization, Offending, Victim–offender Overlap and Parental Violence

As explained above, we conducted multinomial logistic regressions for all violence types combined and each violence type separately (except digital violence). We report the effects using both unstandardized regression weights (*B*) and odd’s ratios (OR) as a standardized and more intuitive effect size. The demographic control variables largely showed no statistically significant association with IPV involvement. For IPV across all types as well as each IPV type, age showed a statistically significant effect on at least one kind of IPV involvement. However, all effects were extremely small and close to zero (−.05 < *B*’s < .03), so we do not discuss them in detail.

Regarding *IPV across all types*, witnessing violence between parents increased the probability/odds of later being victimized by IPV (*B* = .58; OR = 1.78, *p* < .05) and of being both victim and offender (*B* = .31; OR = 1.36, *p* < .05). Verbal parental violence increased the probability/odds of all types of IPV involvement (victim: *B* = .47, OR = = 1.60, *p* < .05; offender: *B* = .68, OR = 1.98, *p* < .05; victim–offender: *B* = .55, OR = 1.78, *p* < .05).

Regarding *physical IPV involvement*, having been victimized by verbal parental violence increased the probability/odds of later being a victim-only (*B* = .37; OR = 1.44, *p* < .05), while witnessing violence between parents (*B* = .36, OR = 1.43, *p* < .05) and having been victimized by physical parental violence (*B* = .86; OR = 2.37) increased the chance of being both a victim and offender.

Parental violence also affected the involvement in *psychological IPV* in multiple ways. Witnessing violence between parents and having been verbally victimized by parents both increased the probability/odds of being an offender-only (*B* = .58, OR = 1.78, *p* < .01 and *B* = .48, OR = 1.44, *p* < .05) and victim–offender (*B* = .29, OR = 1.34, *p* < .05 and *B* = .40, OR = 1.49, *p* < .05). However, having been victimized by physical parental violence increased the probability/odds of only being a victim-only of psychological IPV (*B* = .52, OR = 1.69, *p* < .05).

Regarding *sexual IPV involvement*, verbal parental violence increased the victim-only probability/odds (*B* = .55, OR = 1.68, *p* < .05), and having been victimized by physical parental violence increased the offender-only probability/odds (*B* = .62, OR = 1.73, *p* < .05).

Regarding *coercive control involvement*, witnessing violence between parents increased the probability/odds of later being an offender-only (*B* = .40, OR = 1.49, *p* < .05), and having been victimized by verbal parental violence increased the probability/odds of later being a victim-only (*B* = .36, OR = 1.44, *p* < .05).

## Discussion

In this article, we presented data on IPV victimization, perpetration and victim–offender overlap in a sample of 1,209 men aged 18 to 69 from Germany. Overall, we found substantial lifetime prevalences of IPV *victimization* ranging from 5.4% (sexual violence) to 39.8% (psychological violence) and 12-month prevalences ranging from 2.8% (digital violence) to 25.1% (coercive control) in our sample. Less severe violent acts occurred more often than more severe violent acts, and, for the most part, violence only occurred rarely within the last year. The sample’s corresponding lifetime prevalences of IPV *offending* ranged between 2.3% (digital violence) and 33.4% (psychological violence). Across IPV types, there was a large overlap between victims and offenders (39.5%), particularly for non-physical types of IPV (psychological: 23.6%; coercive control: 20.3%). Offending only was most frequently reported for coercive control (18.4%) and psychological violence (9.8%). For sexual and digital violence, the offending rates were clearly larger than the victimization rates and the victim–offender overlap, albeit on a lower absolute level. Overall, verbal parental violence was the most predictive type of parental violence, as it showed the most associations with IPV involvement. For example, across the different IPV types, verbal parental violence was consistently associated with victimization. Both being victimized by verbal parental violence and witnessing violence between parents predicted perpetrating psychological violence and being a victim–offender of psychological violence. Having been victimzed by physical parental violence in childhood more than doubled the odds of later being a victim–offender of physical IPV, and increased the odds of later becoming an offender-only of sexual IPV and a victim-only of psychological IPV.

These high sample prevalences are consistent with findings in the literature that show that IPV is also a relevant issue for men (Scott-Storey et al., 2022). In line with previous studies, we found that especially non-physical types of violence, that is, psychological violence and coercive control, are reported with substantive frequencies (Dim & Elabor-Idemudia, 2018; Jud et al., 2023). The physical violence rate in our sample was also high, with lifetime prevalences almost reaching 30%. This was considerably more than the 10.8% lifetime prevalence Jud et al. (2023) found in another representative IPV survey from Germany, but much closer to a smaller study on IPV against men in Germany (25%; Jungnitz et al., 2007).

However, the comparability of the current study to Jud et al. (2023) may be limited for several reasons. Apparently, Jud et al. (2023) did not exclude men with no relationship experience as it was done in the current study. As having had a relationship is a logical predisposition for IPV experience, this may have deflated Jud et al.’s (2023) IPV rate, especially since their sample included participants from the age of 14. Also, we used a more comprehensive assessment instrument with eleven items on physical violence, while Jud et al. (2023) assessed it with only three items, and it is plausible to assume that asking about more events tends to increase prevalence rates. However, there was a similar difference between the assessment instruments of psychological violence (ten items vs. five items), yet the lifetime prevalence we found was lower than what Jud et al. (2023) found (39.8% vs. 48%). Thus, more comprehensive measures do not necessarily inflate prevalence rates. Finally, while the sample sizes of both studies were similar (*N* = 1,347 vs. *N* = 1,209), the response rate of the current study was much lower (10.7% vs. 44.1%), and there were indicators of a higher income and higher education level in our sample in our sample (Jud et al., 2023 report no further information about sample representativeness). This may indicate a non-response/self-selection bias in our sample (Draugalis et al., 2008; see more detailed discussion below). Whether this has influenced our results, however, remains unclear, as studies found no straightforward correlation between IPV and socio-economic status (Ahmadabadi et al., 2020; Renner & Whitney, 2010).

While the lifetime prevalence rates in our sample seem very high, the results on the 12-month prevalences help to put them into perspective. The large majority of IPV victims reported that IPV occurred only very rarely within the last 12 months. Explorative analyses also revealed that milder acts of violence were much more frequently reported than more severe acts of violence. Both findings are in line with current studies that show that men are predominantly victimized by relatively rare and less severe acts of violence (Fanslow et al., 2023; Follingstad & Rogers, 2013; Jud et al., 2023). This is not to say that men do not suffer severe IPV with serious consequences; there is ample evidence that they do (Randle & Graham, 2011; Schemmel et al., 2024). However, there is a wide range of violent acts with different severity levels. This should be kept in mind when interpreting prevalence rates of violence types, not actions.

Disentangling victim-only and offender-only rates from the victim–offender overlap helped in drawing a differentiated picture of IPV experiences and should thus be mandatory in studies on IPV. We replicated the well-documented finding that there is a considerable victim–offender overlap in the IPV involvement of men (Richards et al., 2016; Varlioglu & Hayes, 2022) and that this also applies to men in Germany (Clemens et al., 2023). According to SLT, one important reason for this may be that violently socialized individuals not only learn to use violence themselves but also self-select into violent peer-groups and relationships as they normalize violent behavior. However, based on the current dataset, it remains unclear whether the significant overlap between victims and offenders can best be explained by the occurrence of reciprocal violence within relationships, a sequential pattern of victimization and offending in distinct relationships, or a combination of both. Moreover, we lack specific details regarding the potentially offensive or defensive nature of this overlap, as violence may be unprovoked or used as a means of self-defense against attacks (Langhinrichsen-Rohling et al., 2012). Still, our findings contribute to an expanding body of literature suggesting that, akin to other forms of crime, a substantial victim–offender overlap challenges a simplistic categorization of individuals strictly as victims or offenders (Richards et al., 2016; Tillyer & Wright, 2013). Future research endeavors could benefit from collecting more nuanced details on the specific dynamics of violent acts, such as whether they occurred reciprocally during a confrontation or if they were one-sided (Charles et al., 2011).

Separating the victim, offender and victim–offender overlap rates also allowed us to report offender-only rates. On an absolute level, they were comparable to those found by Clemens et al. (2023) for psychological violence (9.8% vs. 9.1%) and sexual violence (6.8% vs. 9.1%), but they were higher for physical violence (5.4% vs. 1.9%). However, the relative differences within each type of IPV pointed in the same direction. In both studies, psychological and physical offending rates were much lower than victimization rates. Also, both studies replicated the consistent findings that men commit sexual violence more frequently than women, and women are overall more frequently victimized than men (Krahé et al., 2021). Notwithstanding the large overlap rates, the offending rates we found also suggest that while victimization seems more common for physical and psychological violence, men who only offend are more likely to engage in coercive control and, even more so, sexual and digital violence. This emphasizes the need for studies to move beyond merely reporting IPV victimization rates, and to always incorporate information on offending as well.

In our study, verbal parental violence was the most important predictor of IPV involvement. First, it significantly increased the odds of IPV victimization regarding physical, sexual, and coercive violence. Thus, growing up with verbally aggressive parents seems to be statistically associated with having violent partners in adulthood. Strikingly, these associations occurred with the IPV types that contain a relatively high share of more severe violent acts. It may be that growing up in verbally but not physically abusive surroundings increases the acceptance of violence against oneself, which makes one vulnerable to more severe IPV. Such an interpretation is in line with SLT and seems more appropriate than a genetic explanation as it is not straightforward why shared genetic predispositions between children and violent parents would increase the association of being victimized as a child and as an adult. Milletich et al. (2010) reported similar results. They found that among adolescent men, dating victimization was predicted by emotional violence in childhood. However, they also found that emotional violence predicted dating aggression among men, while our data showed no increased propensity to engage in physical, sexual, or coercive violence.

In contrast, being victimized by verbal parental violence did increase psychological IPV offending and the victim–offender overlap. Witnessing violence between parents had the same effect (for similar findings, see Roberts et al., 2010). Witnessing violence also increased later being a victim and offender of physical violence. One explanation for this finding may be the genetic similarity between parents and their children. From an SLT perspective, however, overall violent conflict management in childhood homes—both between parents and between parents and children—may increase the odds of men modeling verbally abusive behavior and being involved in physically violent relationships (Akers & Jennings, 2009). It may also promote normalization (Edleson, 1999) and desensitize them to the negative consequences of physical and psychological violence in general, especially because psychological violence typically does not leave visible traces. Consequently, they may not only be violent themselves, but they may also experience IPV victimization, either when the partner reacts with violence themself or because of a differential association with violence-supporting people. Moreover, being physically abused by parents more than doubled the participants’ odds of later offending and being victimized by physical IPV. This is also expected by SLT, as it might indicate that children adopt physically violent behavior and, as adults, get caught in a cycle of violence with partners either fighting back or being proactively violent themselves (Akers & Jennings, 2009). Again, an alternative explanation might be that both children and parents have a genetic predisposition to violence. The finding that physical abuse in childhood increased the odds of sexual IPV offending by 87% suggests that physical parental violence is an unspecific risk factor for hands-on IPV in general.

Overall, our findings deviate from those presented by Varlioglu and Hayes (2022) in a comparable study. In line with meta-analytic findings (Li et al., 2019), we found strong associations between men’s childhood experiences of physical parental violence and later IPV involvement as victim–offenders of physical violence, whereas they found an association between physical abuse and being a victim-only of physical IPV, and no effect on being victim-offender of physical violence. However, Varlioglu and Hayes (2022) differed in their methodology: We based our study on a population-wide sample and used the sample’s lifetime prevalences to calculate associations between parental violence and (phyiscal) IPV, whereas Varlioglu and Hayes (2022) analyzed 12-month prevalences of a college sample.

Our study has several advantages. First, we add to the literature by providing a large data set on IPV against men collected with a representative sampling method that is not only informative regarding the situation in Germany but also helps to further the international discussion about IPV against men. Moreover, our reporting on sample prevalences of both victimization and offending enabled us to disentangle victims, offenders, and victim–offenders, thus painting a more nuanced picture of the reality of IPV. In addition, we used a comprehensive catalog of violent acts that allowed for a fine-grained measurement of IPV.

However, some limitations should be mentioned: First, the low response rate, as well as the slight overrepresentation of men with higher income and level of education, restricts the representativeness of the sample (see above). The reasons for the low response rate remain unknown. Men’s response rates are generally low, especially in online surveys (Becker, 2022). Instead of IPV experiences, the survey invitation stressed that it was about conflicts in relationships. However, studies indicate that men are generally more reluctant to discuss emotions, distress, and personal issues (Addis & Mahalik, 2003; Wagner & Reifegerste, 2024), which still may have discouraged some men from participating. Thus, methodological approaches as chosen by Jud et al. (2023) may be more suitable for IPV studies with men. They embedded questions on IPV and emotional topics in a broader research project not focused on men and had participants fill in questionnaires on sensitive topics after a personal interview and with the researcher staying nearby. This may prevent men from non-participation or canceling their participation. Also, low response rates may be associated with self-selection biases, that is, men with IPV experiences may have participated to a larger extent than men with no IPV experiences. As already explained, we attempted to avoid such biases by labeling the study’s focus as “conflict in relationships.” However, IPV was mentioned casually in the invitation text as a rather extreme form of conflict solution. Also, victims of IPV may be drawn even to a more general topic such as relationship conflicts. Even though the prevalence rates of the current sample overall fit into the empirical picture of IPV against men (see above), a self-selection bias can therefore not be ruled out. Given the potential limitations in the representativeness of our sample and the observed differences, particularly in the frequency of physical violence compared to Jud et al. (2023), the prevalence of IPV against men in Germany requires further investigation.

Moreover, our measurement of violence referred to the experience with single acts; thus, we do not have the information necessary to answer important questions concerning specific circumstances of violence or reciprocity within relationships. Similarly, we cannot reconstruct the sequential order of violence in the victim–offender group. It thus remains an open question how many men started the violence (Hamberger & Larsen, 2015) and how many were fighting back (for a similar discussion, see Jud et al., 2023). Another limitation is that our study relies on subjective and retrospective reports from one partner of a couple. Previous research has underscored the possibility of women underreporting IPV victimization and men underreporting offending, both of which may limit the reliability of our data (Chan, 2011). For example, the men in our sample reported a lifetime prevalence of forced intercourse of 1.3% as victims and 0.6% as perpetrators, which does not seem plausible at first glance (Hamberger & Larsen, 2015); for a thorough review of male sexual victimization, however, see Thomas and Kopel (2023). Also, the offending only rate of physical IPV was quite low (5.4 %; see Jud et al. (2023) for an even lower rate). Moreover, taking only one perspective into account ignores the fact that many of the relevant acts happen in ambiguous and highly emotional situations. In such situations, misunderstandings may occur, and partners may interpret their own actions differently than the other one (Kuijpers, 2020). Thus, couples may have largely divided opinions on IPV, which does not invalidate one partner’s perspective but stresses the complexity of IPV experiences. Also, prevalence rates on distinct violent acts only provide a rough overview and do not sufficiently assess the psychological reality of violent experiences. For example, being screamed at or insulted may, under some circumstances, have little effect on the victim, while under different circumstances, it may severely impact the victim (e.g., when a man is degraded in front of his children). Therefore, large studies such as this one should be complemented by in-depth studies on the subjective experiences of those affected by IPV. Also, the prevalence of sexual and digital violence was very low. Digital violence even lacked appropriate statistical power for inferential analyses. Therefore, the inferential results regarding sexual violence are regarded as too unreliable to draw conclusions upon. Furthermore, our data collection period was four months. During that time, changes in support systems or public opinions may have occurred which may have influenced the results. Finally, our findings regarding associations between parental violence and IPV are purely observational. Especially the associations between being victimized by parents as a child and perpetrating IPV as an adult could be explained by SLT as well as genetical factors. We did not collect the data to rule out either explanation.

## Conclusion

IPV against men is substantively prevalent in Germany, though many violent acts are less severe. More counseling centers specialized in men’s issues are needed, proactively reaching out to encourage men to overcome internal barriers to seeking help. Additionally, reducing harmful male stereotypes requires raising awareness not only in the general public but also within educational institutions and among police officers. This should be a key objective for policymakers and practitioners. As only a minority of men involved in IPV are victims only, most men involved in (serious) IPV need not only victim support but also help as offenders to break the cycle of violence. A rigid classification of individuals as either perpetrators or victims may obscure the view of the nuances underlying domestic violence. Consistent with prior research, our results highlight the significance of parental violence (especially verbal) in predicting IPV involvement, suggesting that early interventions for violence prevention within families could be especially advantageous in this context.

## Supplemental Material

sj-docx-1-jiv-10.1177_08862605251321003 – Supplemental material for Intimate Partner Violence Against Men in Germany—A Study on Prevalence, Victim–Offender Overlap, and the Role of Parental Violence Supplemental material, sj-docx-1-jiv-10.1177_08862605251321003 for Intimate Partner Violence Against Men in Germany—A Study on Prevalence, Victim–Offender Overlap, and the Role of Parental Violence by Jonas Schemmel, Dario Maciey and Laura-Romina Goede in Journal of Interpersonal Violence

## Notes

1. This process was stratified based on region and municipality size using the Cox algorithm, ensuring the inclusion of both rural and urban communities and covering the entire geographical area of Germany. However, due to the anonymous nature of the survey, we were unable to trace participants’ places of residence to analyze regional patterns.

2. Upon request, participants were provided with a paper-pencil questionnaire instead. Fifteen men asked for the paper-pencil version and returned it filled out via mail. These questionnaires were digitized separately and then integrated with the data from the online survey.

3. In the GESIS sample of men aged between 18 and 69, the mean age was 47.53 (*SD* = 14.4), 90% of the participants were born in Germany, 69% fully employed, 21% not employed (including retired and unemployed), 5% were working part-time, 58.5% were married, 32.2% unmarried, 7.5% divorced, 1.1% widowed.

4. We will stick to the term “prevalence” although strictly speaking, it refers to a population, and as explained under “Sample Characteristics,” the representativeness of the sample may be restricted by an overrepresentation of men with higher income and level of education.

5. To test the predictors of the regression models for collinearity, we calculated the variance inflation factors (VIF). The highest VIF is around 2.6, so there is no indication of multicollinearity.
