# Supplementary material for: Intimate Partner Violence Against Men in Germany—A Study on Prevalence, Victim–Offender Overlap, and the Role of Parental Violence
Source: J Interpers Violence. 2025 Feb 26;41(9-10):1686–712. doi: 10.1177/08862605251321003 (PMC13047224; doi:10.1177/08862605251321003)
Supplement: sj-docx-1-jiv-10.1177_08862605251321003 – Supplemental material for Intimate Partner Violence Against Men in Germany—A Study on Prevalence, Victim–Offender Overlap, and the Role of Parental Violence [file sj-docx-1-jiv-10.1177_08862605251321003.docx]

# **Appendix**

| Appendix Table 1.  *IPV Items* | | | | | | | |
| --- | --- | --- | --- | --- | --- | --- | --- |
|  | **Lifetime (victim.)** | **Lifetime (offend.)** | **12-month (victim.)** | **1-2 times** | **3-12 times** | **multiple times/month** | **at least once/week** |
| **Physical Violence** | **29.8%** | **18.9%** | **13.8%** |  |  |  |  |
| Intentionally pushing | 17.6% | 8.9% | 8.7% | 6.3% | 2.0% | 0.1% | 0.0% |
| Light slap in the face | 11.1% | 3.7% | 4.1% | 3.0% | 0.8% | 0.2% | 0.0% |
| Biting, scratching or pinching | 10.0% | 1.8% | 5.2% | 3.5% | 0.9% | 0.4% | 0.0% |
| Grabbing hard, holding on or twisting arms | 8.5% | 8.6% | 3.8% | 2.4% | 1.1% | 0.2% | 0.0% |
| Kicking, violent slapping, hitting the other person | 6.1% | 0.6% | 2.6% | 1.8% | 0.5% | 0.2% | 0.1% |
| Choking or otherwise attempting to suffocate the other person | 0.8% | 0.1% | 0.5% | 0.3% | 0.0% | 0.0% | 0.0% |
| Throwing an object at the other person | 13.3% | 4.7% | 4.8% | 3.4% | 1.1% | 0.1% | 0.0% |
| Attacking the other person with an object (e.g., a pan or a broom) or a weapon (e.g., a knife) | 2.5% | 0.3% | 1.1% | 0.7% | 0.3% | 0.1% | 0.0% |
| Injuring the other person with an object or weapon | 0.9% | 0.0% | 0.3% | 0.2% | 0.0% | 0.1% | 0.0% |
| Intentionally scalding or inflicting burns | 0.2% | 0.0% | 0.0% | 0.0% | 0.0% | 0.0% | 0.0% |
| Confinement or restraint | 1.1% | 0.5% | 0.4% | 0.3% | 0.1% | 0.1% | 0.1% |
| **Psychological Violence** | **39.8%** | **33.4%** | **23.6%** |  |  |  |  |
| Aggressively shouting, abusing or insulting the other person | 33.9% | 27.1% | 20.1% | 10.7% | 5.7% | 2.6% | 0.3% |
| Ridiculing, humiliating or exposing the other person in front of other people | 14.8% | 7.7% | 7.7% | 4.8% | 1.8% | 0.6% | 0.1% |
| Humiliating, devaluing or belittling | 17.0% | 10.4% | 9.7% | 4.1% | 3.3% | 1.3% | 0.4% |
| Intentionally destroying objects or tormenting pets | 7.4% | 3.0% | 2.8% | 1.5% | 1.0% | 0.0% | 0.0% |
| Threatening to harm yourself in order to put pressure on the other person | 10.6% | 1.8% | 3.9% | 2.4% | 1.1% | 0.0% | 0.0% |
| Threatening to take the children away | 5.8% | 0.2% | 2.6% | 1.5% | 0.8% | 0.2% | 0.0% |
| Threatening to harm the children or other loved ones | 1.1% | 0.1% | 0.4% | 0.3% | 0.1% | 0.0% | 0.0% |
| Threatening to harm the other person | 2.3% | 0.7% | 0.8% | 0.2% | 0.4% | 0.1% | 0.1% |
| Seriously threatening the other person with death | 0.5% | 0.2% | 0.2% | 0.2% | 0.0% | 0.0% | 0.0% |
| Threatening with a weapon (e.g., knife) or household object (e.g., frying pan) | 3.4% | 0.2% | 1.4% | 1.0% | 0.3% | 0.0% | 0.1% |
| **Sexual Violence** | **5.4%** | **9.2%** | **3.4%** |  |  |  |  |
| Touching the other person with sexual intent (e.g., kissing, fondling, grabbing) even though the person has said or indicated that they do not want it | 4.6% | 8.4% | 2.5% | 1.6% | 0.7% | 0.1% | 0.0% |
| Forcing the other person to sexually satisfy themselves or the other person with their mouth, hand or object, even though the person has said or indicated that they do not want to | 0.8% | 0.8 | 0.3% | 0.1% | 0.0% | 0.0% | 0.0% |
| Attempting to penetrate the other person's body with a finger or object even though the person has said or indicated that they do not want it (but it did not happen) | 0.5% | 0.7% | 0.5% | 0.2% | 0.3% | 0.0% | 0.0% |
| Penetrating the other person's body with a finger or an object even though the person has said or indicated that they do not want it | 0.3% | 0.6% | 0.1% | 0.1% | 0.1% | 0.1% | 0.0% |
| Trying to force the other person to have sex/intercourse even though the person has said or indicated that they do not want to (but it did not happen) | 1.3% | 0.6% | 0.9% | 0.6% | 0.3% | 0.0% | 0.1% |
| Forcing the other person to have sex/intercourse even though they have said or indicated that they do not want to | 1.0% | 0.6% | 0.7% | 0.3% | 0.3% | 0.0% | 0.1% |
| **Coercive Control** | **38.6%** | **31.5%** | **25.1%** |  |  |  |  |
| Prescribing or controlling how much money the other person spends | 11.0% | 11.1% | 7.3% | 3.2% | 2.3% | 1.5% | 0.1% |
| Preventing or controlling the other person's contact with family, friends or the other person's activities outside the home | 16.6% | 3.8% | 7.7% | 3.9% | 3.5% | 1.0% | 0.2% |
| Repeatedly following, spying on or lying in wait for the other person | 6.1% | 2.4% | 3.2% | 1.4% | 1.3% | 0.4% | 0.1% |
| Massive intimidation if the other person disagrees (e.g., through gestures, looks or shouting) | 17.2% | 10.3% | 9.2% | 3.9% | 5.6% | 3.3% | 0.6% |
| Checking the other person's mail, chat history, emails or phone calls | 15.6% | 9.7% | 8.1% | 3.6% | 1.6% | 1.6% | 0.2% |
| Saying that the other person couldn't cope without you | 10.0% | 6.4% | 5.7% | 2.6% | 1.9% | 0.5% | 0.2% |
| Blaming the other person for everything and constantly making the other person feel guilty | 23.4% | 10.6% | 14.7% | 3.8% | 2.2% | 1.3% | 0.3% |
| **Digital Violence** | **6.5%** | **2.3%** | **2.8%** |  |  |  |  |
| Spreading personal information or nasty rumors about the other person on social networks/the internet | 3.2% | 0.1% | 1.2% | 0.5% | 0.3% | 0.1% | 0.0% |
| Monitoring the other person (e.g., using a camera or an app on their cell phone) | 2.2% | 1.4% | 1.1% | 0.5% | 0.5% | 0.1% | 0.0% |
| Publishing intimate or embarrassing pictures or videos of the other person online | 0.9% | 0.1% | 0.4% | 0.3% | 0.0% | 0.0% | 0.0% |
| Intimidating, blackmailing or threatening the other person through emails or text messages | 4.2% | 0.8% | 1.8% | 0.6% | 0.5% | 0.6% | 0.0% |
| *Note.* This table provides the wordings of the IPV acts included in the current study, not how exactly these items were presented to the participants (for more information on that, please refer to the Method section). The categories “1-2 times”, “3-12 times”, “multiple times/month”, “at least once/week” all refer to the last 12 months. The categorie “at least once/week” summarizes “once a week”, “multiple times per week”, and “daily”. | | | | | | | |
